# High Pressure Processing Impact on Emerging Mycotoxins (ENNA, ENNA1, ENNB, ENNB1) Mitigation in Different Juice and Juice-Milk Matrices

**DOI:** 10.3390/foods11020190

**Published:** 2022-01-12

**Authors:** Noelia Pallarés, Albert Sebastià, Vicente Martínez-Lucas, Rui Queirós, Francisco J. Barba, Houda Berrada, Emilia Ferrer

**Affiliations:** 1 Preventive Medicine and Public Health, Food Science, Toxicology and Forensic Medicine Department, Faculty of Pharmacy, Universitat de València, Avda. Vicent Andrés Estellés, Burjassot, 46100 València, Spain; noelia.pallares@uv.es (N.P.); alsedu@alumni.uv.es (A.S.); marluvi@alumni.uv.es (V.M.-L.); emilia.ferrer@uv.es (E.F.); 2 Hiperbaric, S.A., C/Condado de Treviño, 6, 09001 Burgos, Spain; r.queiros@hiperbaric.com

**Keywords:** high pressure processing, thermal treatment, enniatins, juice models, dispersive liquid-liquid microextraction methodology (DLLME), liquid chromatography coupled to ion-trap tandem mass spectrometry (HPLC-MS/MS-IT)

## Abstract

The aim of the present study was to investigate the potential of high-pressure processing (HPP) (600 MPa during 5 min) on emerging mycotoxins, enniatin A (ENNA), enniatin A1 (ENNA1), enniatin B (ENNB), enniatin B1 (ENNB1) reduction in different juice/milk models, and to compare it with the effect of a traditional thermal treatment (HT) (90 °C during 21 s). For this purpose, different juice models (orange juice, orange juice/milk beverage, strawberry juice, strawberry juice/milk beverage, grape juice and grape juice/milk beverage) were prepared and spiked individually with ENNA, ENNA1, ENNB and ENNB1 at a concentration of 100 µg/L. After HPP and HT treatments, ENNs were extracted from treated samples and controls employing dispersive liquid-liquid microextraction methodology (DLLME) and determined by liquid chromatography coupled to ion-trap tandem mass spectrometry (HPLC-MS/MS-IT). The results obtained revealed higher reduction percentages (11% to 75.4%) when the samples were treated under HPP technology. Thermal treatment allowed reduction percentages varying from 2.6% to 24.3%, at best, being ENNA1 the only enniatin that was reduced in all juice models. In general, no significant differences (*p* > 0.05) were observed when the reductions obtained for each enniatin were evaluated according to the kind of juice model, so no matrix effects were observed for most cases. HPP technology can constitute an effective tool in mycotoxins removal from juices.

## 1. Introduction

Mycotoxins are secondary metabolites produced naturally by certain filamentous fungi that can be found, usually at low concentrations, in a wide range of food matrices such as cereals, nuts, herbal teas, wine, coffee or species. *Aspergillus*, *Penicillium*, *Alternaria*, *Fusarium*, and *Claviceps* genus constitute the main mycotoxins producers [1,2]. Mycotoxin exposure via food and feed may produce several adverse health effects, such as carcinogenicity, immunotoxicity, reproductive toxicity, hepatotoxicity, and nephrotoxicity [3]. Among 300 mycotoxins identified, only aflatoxins (AFs), trichothecenes (TCs), zearalenone (ZEA), fumonisins (FBs), ochratoxins (OTs), and patulin (PAT) are regularly analyzed in food. Although emerging mycotoxins have not been regulated yet, and maximum levels have not been established in food products, a growing interest has been shown in evaluating their prevalence in food and feed, as well as their potential toxicity in humans and animals due to their high interest and concern [4,5]. In fruits and their processed products, such as juice beverages, PAT, OTA, and *Alternaria* toxins constitute the most reported mycotoxins [6], however emerging mycotoxins are ubiquitous food contaminants that occur frequently in agricultural products [7].

Enniatins (ENNs) belong to the group of emerging mycotoxins and present a cyclic hexadepsipeptides structure. These mycotoxins are produced by several *Fusarium* species, such as *F. avenaceum*, *F. oxysporum*, *F. poae*, or *F. tricinctum*, which mainly grow on cereals. More than 29 species of ENNs are known, being the most detected in food and feed ENNA, ENNA1, ENNB and ENNB1. The toxicity of ENNs is based on their ionophoric properties. These toxic compounds facilitate the transport of mono and divalent cations across membranes, resulting in a disruption of normal physiological concentrations [8]. Besides their ionophoric properties, these mycotoxins are related to cell damage, such as oxidative stress, mitochondrial modifications and the disruption of the cell cycle inducing several health adverse effects such as genotoxicity, immunotoxicity, neurotoxicity and endocrine toxicity. Over the last few years, an increased number of in vitro and in vivo studies trying to understand the ENNs mechanisms of action have been evaluated [9,10]. In this sense, EFSA (2014) established a lethal dose (LD50) of 350 mg/kg/bw in mice upon oral administration for a mixture of ENNs. The cytotoxicity studies regarding their exposure to different cell lines revealed inhibitory concentration values (IC50) from 2.6 to 36 µM [11]. Despite their adverse effects on human and animal health, some beneficial properties have been attributed to ENNs, showing an interesting activity against various microorganisms and insects in several studies, leading to a potential use as biopesticides, as well as anticancer molecules combined with other drugs [12].

In recent years, innovative food processing technologies such as high-pressure processing (HPP), pulsed electric fields (PEFs), ultrasound (US), and cold plasma (CP) have emerged in the food industry addressing specific consumer needs toward safe, healthy, and minimally processed foods. These technologies overcome some limitations of traditional processing practices and constitute an environmentally friendly and sustainable food manufacturing techniques alternative [13]. Moreover, these processes can better retain biologically active compounds present in food and organoleptic characteristics [14,15].

HPP technology is based on two fundamental principles, the Le Chatelier and the isostatic principles. The HPP treatment is characterized mainly by the temperature, pressure, and exposure time parameters. In food preservation, the pressures employed are in the range between 400 and 600 MPa. These pressures are applied during short periods (several seconds to minutes) at cold or mild temperatures (4–25 °C) [16]. This technology facilitates the inactivation and inhibition of microorganisms, and it can also activate or inactivate enzymes at low temperatures, while low molecular weight compounds involved in the flavor and nutritional value remain unaltered [17]. More recently, some authors have investigated the potential role of innovative food processing technologies in pesticides and mycotoxins removal, concluding that the efficiency depends on different factors, such as the processing parameters, the type of pesticide/mycotoxin and the food matrix. Thus, variable reduction percentages (from 0 to 100%) have been reported by several authors in food matrices, such as fruit and vegetable beverages, maize and olives, depending on the level of contamination, temperature, treatment time and pressure applied [18,19,20,21,22].

Most mycotoxins are moderately heat-stable. However, various industrial food processes, such as cleaning, sorting, trimming, milling, brewing, cooking, baking, frying, roasting, canning, flaking, alkaline cooking, nixtamalization, and extrusion, may have a variable impact on mycotoxins, resulting from those which utilize high temperatures in greatest effects. For instance, roasting and extrusion cooking at high temperatures (above 150 °C) appear to reduce mycotoxin concentrations [23,24]. The reduction achieved depends on several factors, such as the nature and chemical structure of the mycotoxins, the initial level of mycotoxin contamination and factors related to the treatment applied (temperature, time, pH). Regarding emerging mycotoxins, although limited data are available on the effects of food processing on ENNs contents, several authors have suggested that ENNs contents are reduced through common cooking and industrial processes such as pasta boiling [25], fish cooking (oven, microwave, broiled and boiled treatments) [26], beer-making [27] and bread-making [28].

In a previous study carried out by our research team, the use of pulsed electric fields (PEF) promoted the reduction of ENNs from 43 to 70% in juice and smoothie beverages, respectively [29], however, to the best of our knowledge there is no information available regarding the impact of HPP on ENNs mitigation in this kind of beverages.

The behavior of mycotoxins is strongly influenced by the matrix, their chemical structure, hydrophobicity, and thermal-mechanical susceptibility. Thus, food processing can result in mycotoxins binding with several matrix components, such as proteins or starch, through the formation of covalent adducts [30]. Thus, detailed information about the fate of mycotoxins during food processing is imperative for a correct risk assessment.

The aim of the present work was to investigate the potential of HPP technology on emerging mycotoxins reduction in different juice/milk beverages, exploring the possible effect of matrix components on the results obtained. Moreover, the results have been compared with those obtained by the traditional thermal treatment.

## 2. Materials and Methods

### 2.1. Reagents and Chemicals

Reagents and chemicals employed are described in detail in a previous work [31].

Methanol (MeOH) and acetonitrile (ACN), (HPLC grade) and Chloroform (CHCl3) (99% grade) were purchased from Merck (Darmstadt, Germany). Ethyl acetate (EtOAc) (HPLC grade 99.5+%) was supplied by Alfa Aesar (Karlsruhe, Germany). Deionized water (resistivity > 18 MΩ cm^−1^) was obtained from a Milli-Q SP^®^ Reagent Water System (Millipore Corporation, Bed-ford, MA, USA). Ammonium formate salt (99%) was obtained from Panreac Quimica S.A.U. (Barcelona, Spain) and sodium chloride (NaCl) was supplied by VWR Chemicals (Leuven, Belgium). Formic acid (reagent grade ≥ 95%) was supplied by Sigma-Aldrich (St. Louis, MO, USA). Enniatin standards (ENNA, ENNA1, ENNB and ENNB1) were purchased from Sigma (St. Louis, MO, USA).

### 2.2. Sample Preparation

Different beverages made from fruits (orange, strawberry, or grape juices) or combinations of fruit with milk (orange juice/milk, strawberry juice/milk and grape juice/milk beverages) were prepared employing the ingredients detailed in Table S1 according to a previous work [32]. A total volume of 1.5 L was prepared for each studied beverage, several aliquots were taken to test the absence of mycotoxins and finally a volume of 1.2 L was spiked individually at a concentration of 100 μg/L with ENNA, ENNA1, ENNB and ENNB1, respectively. To be employed as untreated controls, several aliquots were taken while the rest of the spiked beverage was bottled per triplicated and refrigerated at 4 °C until the treatment.

### 2.3. HPP Treatment

Samples were treated using high-pressure Hiperbaric 55 equipment (Burgos, Spain). The instrument was equipped with a 55 L pressure chamber. Water was employed as a pressure-transferring medium at 10–12 °C. According to the literature, as a consequence of the adiabatic heating, the temperature of the samples is expected to have increased at a rate of c.a. 3 °C = 100 MPa. The come-up rate was approximately 3.6 MPa/s. To treat the samples, the conditions were set at 600 MPa and 5 min. Prior to the treatment, the bottled samples were placed in plastic bags filled with a solution of hydrogen peroxide and deionized water (1:25). The vacuum was then applied prior to heat sealing.

### 2.4. Thermal Treatment (HT)

In order to simulate conventional juice pasteurization, a thermal treatment (HT) was conducted at 90 °C for 21 s according to a previous work [32]. For this purpose, a Julabo circulating water bath (Seel-bach, Germany) was used. All the experiments were carried out in triplicate. Three aliquots of each juice model were separated to be employed as untreated controls.

### 2.5. DLLME Extraction

DLLME extraction procedure was employed to extract ENNs from treated and control samples according to a previous study [31]. Then, 5 mL of juice beverage was placed in a 10 mL conical tub containing 1 g of NaCl. The tube was then shaken, and a mixture of dispersant solvent (950 μL of AcN) and extractant solvent (620 μL of EtOAc) was added in the first step. After shaking and centrifugating at 4000 rpm for 5 min, the phases were separated, and the organic phase located at the top of the tub was recovered and placed into another conical tube. Then, the mixture of dispersant solvent (950 μL of MeOH) and extractant solvent (620 μL of CHCL3) was added to the remaining residue in a second extraction step and proceeded as detailed before. In this case, the organic phase was located at the bottom of the tube. Both organic phases recovered were evaporated employing a Turvovoap LV Evaporator (Zy-mark, Hoptikinton, MA, USA). Finally, the samples were reconstituted in a vial employing 1 mL of 20 mM ammonium formate (MeOH/ACN) (50/50 *v*/*v*) and filtered through a 13 mm/0.22 μm nylon filter prior to analysis.

### 2.6. LC-MS/MS-IT Determination

An Agilent 1200 chromatograph (Agilent Technologies, Palo Alto, CA, USA) coupled to a 3200 QTRAP^®^ (Applied Biosystems, AB Sciex, Foster City, CA, USA) with Turbo Ion Spray (ESI) electrospray ionization was employed for the determination. The instrumental parameters were fixed as previously indicated [31].

The chromatographic separation was performed with a Gemini-NX column C18 (Phenomenex, 150 mm × 4.6 mm, 5 particle size) preceded by a guard column. Mobile phases consisted of: 5 mM ammonium formate, 0.1% formic acid water (A) and 5 mM ammonium formate, 0.1% formic acid methanol (B). The gradient program started with a proportion of 0% for eluent B; increasing to 100% in 10 min, then decreasing to 80% in 5 min, and finally to 70% in 2 min. Then, the column was cleaned and readjusted to initial conditions in the next 6 min, and equilibrated during 7 min. The instrumental parameters were fixed as follows: injection volume of 20 µL, flow rate at 0.25 mL/min and oven temperature at 40 °C. The Turbo Ion Spray operated in positive ionization mode (ESI+). Nitrogen served as a nebulizer and collision gas. Ion spray voltage was fixed at 5500 V, the curtain gas was set at 20 (arbitrary units), the nebulizer (GS1) and TIS (GS2) gases at 50 and 50 psi, respectively, and the probe temperature (TEM) was set at 450 °C.

### 2.7. Method Validation

The methodology employed in the present study was validated in a previous work [31] following the requirements established by the Commission Decision 2002/657/EC [33]. Recovery experiments were carried out at three contamination levels (50, 100, 200 µg/L) and revealed good recoveries for all ENNs with percentages ranging between 66 and 110%. The intra-day and inter-day precision were lower than 19%. Regarding matrix effect experiments, no signal suppression-enhancement was observed for none of the studied ENNs. The LOD and LOQ obtained for ENNB were 0.15 and 0.5 µg/L, respectively, while 0.3 and 1 µg/L were the LOD and LOQ, obtained for the rest of ENNs (ENNA, ENNA1, ENNB1). Regression coefficients showed good linearity.

### 2.8. Statistical Analyses

All statistical analyses were carried out employing the software GraphPad Prism8.0.2 (GraphPad Software, San Diego, CA, USA). The results were analyzed employing an analysis of variance (3-way ANOVA) followed by Tukey’s test to determine the significance of differences between treatments (HPP and thermal) and juice models and mycotoxin concentrations. A probability value of *p* < 0.05 was considered to be significant.

## 3. Results

### 3.1. Effect of HPP on ENNs Contents in the Studied Juice Models

**Figure 1:**
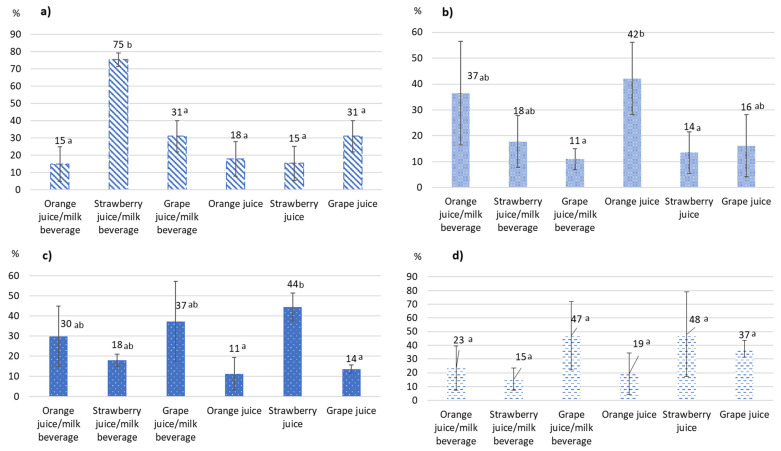
Reduction percentages (%) achieved for (**a**) enniatin A (ENNA), (**b**) enniatin A1 (ENNA1), (**c**) enniatin B (ENNB), and (**d**) enniatin B1 (ENNB1), respectively, in the different juice models studied after high-pressure processing (HPP) (600 MPa during 5 min). Bars with different letters indicate significant statistical differences (*p* < 0.05) between different juice models.

The results obtained revealed a significant (*p* < 0.05) reduction of ENNs after HPP treatment in all juice models studied (orange juice, orange juice/milk beverage, strawberry juice, strawberry juice/milk beverage, grape juice and grape juice/milk beverage) spiked individually with each enniatin at 100 µg/L (Figure 1). After the treatment, the calculated concentrations ranged from 24.6 to 88.9 µg/L, which is equivalent to reduction percentages ranging between 11% and 75.4% (Figure 1, Table 1). According to the kind of ENNs, the reduction percentages obtained ranged from 14.9 to 75.4% for ENNA, 11 to 42.1% for ENNA1, 11.3 to 44.4% for ENNB, and 15.3 to 48.14% for ENNB1, respectively, being the ENNA the one presenting a wider range.

To the best of our knowledge, there are no data available in the literature about the effect of HPP on ENNs mitigation. In the same matrix, several authors have explored the effect of HPP on PAT reduction (Table 2). For instance, Hao et al. [19] obtained a reduction of around 30% in a juice beverage based on romaine, celery, cucumber, apple, spinach, kale, parsley and lemon mixture that was spiked with PAT at 200 μg/L and HPP-treated (600 MPa/5 min/11 °C). Slightly lower reductions (21.5–24.5%) were observed by these authors in other juice formulations (apple and spinach; pineapple, apple and mint; and apple, carrot, beet, lemon and ginger). Likewise, in commercial apple juice contaminated with PAT at 5, 50 and 100 μg/L, Avsaroglu et al. [22] obtained up to 51.16% reduction percentages after the application of HPP (400 MPa/30 °C). Those percentages increased up to 62.11% when HPP was combined with pulses (pulsed-high hydrostatic pressure treatment). The reduction percentages obtained in the present study for emerging mycotoxins were in the range of those obtained by these authors.

Concerning other mycotoxins, in a commercially grape juice, the HPP (500 MPa/5 min) promoted AFs reduction percentages up to 17% for aflatoxin B1 (AFB1), 14% for aflatoxin B2 (AFB2), 19% for aflatoxin G1 (AFG1) and 29% for aflatoxin G2 (AFG2) [34]. These results are also in close agreement with the results obtained in the present study. However, in the present work slightly higher reduction percentages were observed in some cases, possibly due to the higher pressure used (500 vs. 600 MPa).

In other matrices, such as maize, HPP (550 MPa/20 min/45 °C) resulted in a complete DON and ZEA reduction [21]. In olive samples, citrinin (CIT) spiked at 1, 1.25, 2.5, 10, 25, and 100 µg/L, which was reduced as an average of 100%, 98%, 55%, 37%, 9% and 1.3%, respectively, as a consequence of HPP treatment at 250 MPa during 5 min. At low mycotoxins concentration (1 µg/L), these authors reported 100% of CIT inhibition. However, only 1.3% reduction was observed at the concentration studied in the present work (100 µg/L) [18]. Thus, the HPP effect depends on the studied mycotoxin, the food matrix and the applied conditions (Table 2).

To the best of our knowledge, this is the first time that HPP technology has been studied in ENNs mitigation. Thus, the results obtained have been compared to those reported in a previous work [29], where the impact of PEF on ENNs (ENNA, ENNA1, ENNB and ENNB1) reduction from grape juices and smoothies, spiked individually also at a concentration of 100 µg/L, was explored. PEF technology (3 kV/cm and specific energy of 500 kJ/kg) resulted in reduction percentages ranging from 43 to 60%. In the present study, the reduction percentages obtained were in a wider range (11 to 75.4%). However, the percentage of 75.4% was only observed for ENNA in strawberry juice/milk beverage, being the reduction percentages in the rest of the juice models for the other ENNs under 48%. The results suggest that PEF technology seems to be more effective in ENNs mitigation than HPP. This can be attributed to the fact that emerging mycotoxins are relatively sensible to temperature and during PEF application temperatures around 70 °C were reached, whereas during the HPP no more than 42 °C (not measured) were expected as a consequence of the adiabatic heating. Thus, the temperature may contribute to ENNs degradation obtained under PEF treatment.

During food processing, some degradation or modified ENNs products can be generated. Several degradation products of ENNs have been reported in the literature after PEF treatment in juice matrices [29], and thermal treatment of cooking pasta [35] or boiling fish [26]. Most of them correspond to the original molecules with the loss of structural amino acidic fragments such as HyLv, Val or Ile. A large majority of these compounds have not been tested yet for potential health adverse effects, however in general the in silico toxicological predictor tools revealed that these degradation products are less toxic than original precursors. The main objective of in silico tools is to prioritize substances for further in-depth toxicological evaluation, so these methods cannot substitute for the in vitro and in vivo studies. Therefore, more detailed toxicology studies are necessary to confirm this statement.

As depicted in Figure 1, after comparing the reductions obtained for each enniatin per type of juice model studied, no overall significant (*p* > 0.05) differences were observed, independently of the sample evaluated. More concretely, for ENNB1 no significant differences were observed between the different juice models studied, whereas for ENNA, the reduction achieved in strawberry juice/milk beverage (75%) was significantly different (*p* < 0.05) from those obtained in the rest of juice models. In addition, for ENNB, the reduction obtained in strawberry juice (44%) was significantly different from those observed in orange juice (11%) and grape juice (14%). Likewise, for ENNA1 the reduction observed in orange juice (42%) was significantly different from those obtained in grape juice/milk beverage (11%) and strawberry juice (14%). However, in general, no significant differences could be associated with the matrix.

### 3.2. Effect of Thermal Treatment (HT) on ENNs Contents in the Studied Juice Models

**Figure 2:**
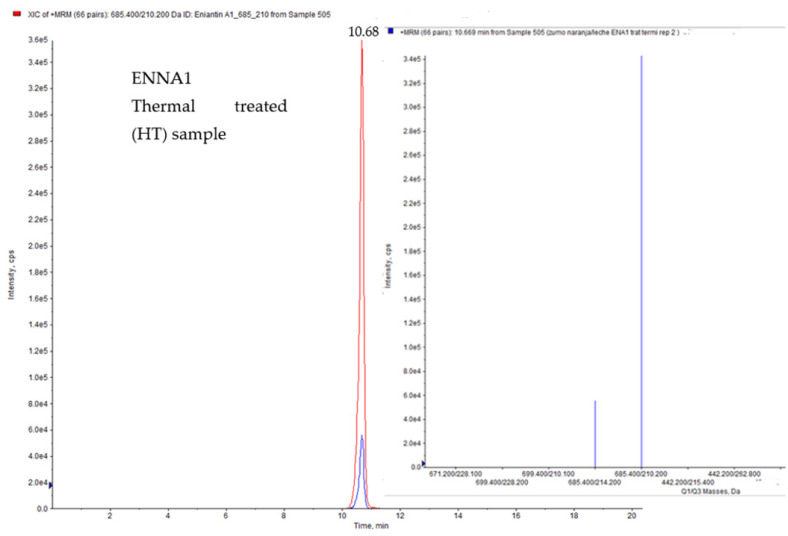

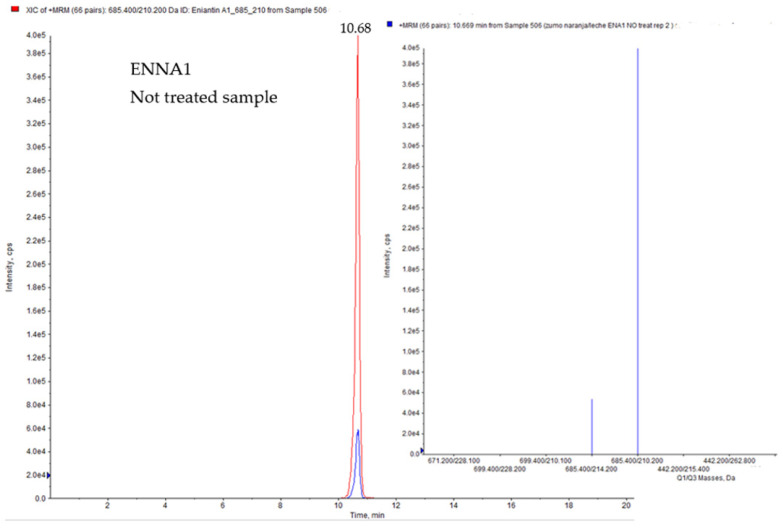
LC-MS/MS-IT chromatogram of orange juice/milk beverage contaminated by ENNA1 (100 µg/L), comparing thermal treated vs. non-treated.

The effect of traditional thermal treatment was also explored in this work in order to compare the results in ENNs mitigation with those obtained after applying HPP. For this purpose, all juice formulations studied in the present work were treated in parallel at 90 °C for 21 s. After the treatment, the calculated concentrations ranged between 75.7 and 100 µg/L (Table 1), so enniatins were only reduced in some cases with percentages ranging from 2.6% to 24.3%. Concretely, a 24.3% reduction was observed for ENNA in the orange juice beverage, significantly different (*p* < 0.05) from the other juice models. For ENNB, 5.9% and 6.1% of reductions were obtained in orange juice and grape juice/milk beverage, respectively, while 2.6%, 10.5% and 9.2% reductions were achieved for ENNB1 in strawberry juice/milk, orange juice and grape juice/milk beverages, respectively. In contrast, ENNA1 was the only one resulting in a reduced amount in all juice models after thermal treatment, with the following percentages of reduction: orange juice/milk beverage (6.0%), strawberry juice/milk beverage (8.7%), orange juice (15.4%), strawberry juice (14.0%), grape juice (6.7%) and grape juice/milk beverage (16.2%). Thus, for ENNA1, ENNB and ENNB1 no significant differences were obtained between the different juice models after the thermal treatment. Figure 2 shows the chromatograms of the orange juice/milk beverage sample spiked with ENNA1, comparing thermal-treated vs. no treated sample. As can be observed, HT did not produce a significant reduction in ENNA1 contents.

Comparing these results with the information available in the literature, higher reduction percentages (up to 100%) were reported by other authors after thermal treatments such as boiling pasta and cooking fish (Table 2). For instance, Serrano et al. [25] observed reduction percentages ranging from 98 to 100% for ENNA, 94–95% for ENNA1, 14–49% for ENNB and 53–65% for ENNB1 after cooking pasta for 10 min in boiling water (100 °C). Contrary to these authors, Nijs et al. [36] observed that cooking pasta under the same conditions hardly affected the contents of ENNs in the end product, reaching only 0–17% of reduction, but in this case, it is important to highlight that the study was performed with naturally contaminated pasta. In another study, Tolosa et al. [26] reported ENNs reductions from 62 to 100% after boiling fish for 5 min, with a temperature in the center of the fillet reaching between 63 and 68 °C. In a previous work, a similar trend was observed. The infusion process of medicinal plants naturally contaminated with ENNs under boiling water at 90–100 °C for 5 min resulted in mean reductions of 95% for ENNB and 100% for the rest of ENNS [37]. The lower reduction percentages observed in the present study (0–24.3%) after thermal processing may be attributed to the fact that the treatment time (21 s) may not be enough to achieve a higher impact on ENNs, as well as the temperature applied (90 °C), was also slightly lower than the boiling temperature employed by other authors. Similarly, the results observed by Serrano et al. [25], ENNs type B seem to be more relatively stable to temperature than type A in the present study.

Comparing the results obtained by both, HPP and thermal treatments, HPP seems to be more effective in ENNs mitigation, due to the higher reductions achieved (11–75.4%). Table 1 shows the ENNs contents observed after HPP and thermal treatments. Moreover, the statistical analysis revealed significant differences (*p* < 0.05) for ENNA in strawberry juice/milk, grape juice/milk and grape juice beverages when thermal treatment and HPP were applied. Moreover, significant differences were also obtained for ENNB contents in strawberry juice and grape juice/milk beverage when both treatments were compared. Finally, in the case of ENNB1, the content achieved after both treatments was only significantly different in strawberry juice. In contrast to those observed with the other ENNs, the contents obtained for ENNA1 after thermal and HPP processes were not statistically significantly different in all the juice formulations studied, maybe because it was the only one where reductions were verified in all formulations after thermal treatment (6–16.2%).

Similar to what was observed in the present study, HPP was also found to be more effective than thermal treatment for mycotoxins reduction (AFB1 and AOH mycotoxins in juice), obtaining a 12% and 7% decrease for AFB1 and AOH, respectively, in grape juice when thermal treatment was applied without reductions obtained in the rest of juice models (orange juice and strawberry juice/milk beverages) [38].

The total enniantin contents in treated juice decreased more after HHP processing than thermal treatment regardless of the juice compounds. These new insights are important considerations in selecting the juice processing method to meet the elevated requirements of quality and provide a comprehensive understanding of the advantages of HHP processing on mycotoxin inactivation in several juice models.

**Table 2:** Mycotoxin reduction percentages reported in the literature after different types of food processing.

Mycotoxin	Type of Matrix	Treatment Conditions	Spiked Concentration/ (% Reductions Achieved)	Reference
HPP treatment				
PAT	Vegetable juices	600 MPa/5 min/11 °C	200 µg/L/ (30%)	Hao et al. [19]
PAT	Apple juice	400 MPa/5 min/30 °C	50 µg/L/ (51.16%)	Avsaroglu et al. [22]
AFs	Grape juice	500 MPa/5 min	100 µg/L/ AFB1 (17%) AFB2 (14%) AFG1 (19%) AFG2 (29%)	Pallarés et al. [34]
ZEA, DON	Maize	550 MPa/20 min/45 °C	Naturally contaminated/ (100%)	Kalagatur et al. [21]
CIT	Olives	250 MPa/5 min	1 µg/L/(100%) 1.25 µg/L/(98%) 2.5 µg/L/(55%) 10 µg/L/(37%) 25 µg/L/(9%) 100 µg/L/(1.3%)	Tokuşoǧlu et al. [18]
PEF treatment				
ENNS	Grape juices and smoothies	3 kV/cm and specific energy of 500 kJ/kg	100 µg/L/ (43–60%)	Pallarés et al. [29]
Boiling				
ENNS	Pasta	10 min/100 °C	0.2–3.5 mg/kg/ ENNA (98–100%) ENNA1 (94–95%), ENNB (14–49%) ENNB1(53–65%)	Serrano et al. [25]
ENNS	Pasta	10 min/100 °C	Naturally contaminated/ (0–17%)	Nijs et al. [36]
ENNS	Fish	5 min/100 °C	Naturally contaminated/ (62–100%)	Tolosa et al. [26]
ENNS	Medicinal plants	5 min/90–100 °C	Naturally contaminated/ ENNB (95%) ENNA, ENNA1, ENNB1 (100%)	Pallarés et al. [37]

## 4. Conclusions

The results obtained in the present study evidenced that HPP technology constitutes an effective tool to decrease enniatin content in juice beverages. HPP treatment (600 MPa/5 min), allowed reduction percentages of enniatins ranging from 11% to 75.4%. In general, no significant differences were observed in enniatins’ contents after HPP independently of the different juice models studied. The thermal treatment, carried out at 90 °C for 21 s, achieved reduction percentages ranging from 2.6% and 24.3% in some cases, being ENNA1 the only enniatin that was reduced in all juice models after thermal treatment. Thus, HPP technology seems to be more effective in removal of enniatins from fruit juices than traditional thermal treatment as it provides higher reduction percentages. Future investigation is necessary to explore the potential of combining HPP with other sustainable technologies and for a better understanding of HPP mechanisms in mycotoxins reduction.

## Figures and Tables

**Table 1:** Contents of enniatins (µg/L) obtained after HPP and HT treatments in the different juice models studied.

Enniatins	Orange Juice/Milk Beverage		Strawberry Juice/Milk Beverage		Grape Juice/Milk Beverage		Orange Juice		Strawberry Juice		Grape Juice	
	HPP	HT	HPP	HT	HPP	HT	HPP	HT	HPP	HT	HPP	HT
ENNA	85.1 ± 10 ^B^	NR	24.6 ± 4 *^A^	NR	69.0 ± 9 *^B^	NR	82.2 ± 10 ^B^	75.7 ± 14 ^C^	84.7 ± 10 ^B^	NR	69.0 ± 9 *^B^	NR
ENNA1	63.5 ± 24 ^AB^	93.8 ± 7	82.2 ± 10 ^AB^	91.3 ± 9	88.9 ± 4 ^A^	83.8 ± 25	57.9 ± 14 ^B^	84.6 ± 1	86.5 ± 8 ^A^	86.0 ± 17	83.8 ± 12 ^AB^	93.3 ± 3
ENNB	70.2 ± 15 ^AB^	NR	81.9 ± 3 ^AB^	NR	62.9 ± 23 *^AB^	93.8 ± 30	88.7 ± 8 ^A^	94 ± 19	55.6 ± 7 *^B^	NR	86.5 ± 2 ^A^	NR
ENNB1	76.7 ± 16	NR	84.7 ± 8	97.4 ± 6	52.9 ± 32	90.8 ± 30	80.8 ± 15	89.5 ± 7	51.9 ± 31 *	NR	62.6 ± 6	NR

Note: The values were expressed as mean± standard deviation (SD). NR indicates no reduction after treatment, contents are approximately 100 µg/L (the initial spiked level). * Indicates that contents obtained after HPP processing are significantly different (*p* < 0.05) from those obtained by HT. Different letters (A, B, C) between different juice models show significant differences (*p* < 0.05) between juice models per each enniatin and treatment.

## Data Availability

Not applicable.

## Supplementary Materials

The following supporting information can be downloaded at: https://www.mdpi.com/article/10.3390/foods11020190/s1, Table S1: Ingredients employed (per 100 mL) to prepare the different juice/milk beverages.

## Sample Availability

Samples of the compounds are available from the authors.
