# Supplementary material for: High Pressure Processing Impact on Emerging Mycotoxins (ENNA, ENNA1, ENNB, ENNB1) Mitigation in Different Juice and Juice-Milk Matrices
Source: Foods. 2022 Jan 12;11(2):190. doi: 10.3390/foods11020190 (PMC8774803; doi:10.3390/foods11020190)
Supplement: Supplementary file 1 [file foods-11-00190-s001.zip › foods-1482887-supplementary.pdf]

Table S1. Ingredients employed (per 100 mL) to prepare the different juice/milk beverages.

Ingredients	Orange Juice	Strawberry Juice	Grape Juice	Orange Juice/Milk Juice	Strawberry Juice/Milk Juice	Grape Juice/Milk Juice
Juice	70 mL	50 mL	60 mL	50 mL	30 mL	50 mL
Skim milk	0 mL	0 mL	0 mL	20 mL	20 mL	20 mL
Bottled water	30 mL	50 mL	40 mL	30 mL	50 mL	30 mL
Pectin	0.3 g	0.3 g	0.3 g	0.3 g	0.3 g	0.3 g
Sugar	7.5 g	7.5 g	7.5 g	7.5 g	7.5 g	7.5 g
Citric acid	0.1 g	0.1 g	0.1 g	0.1 g	0.1 g	0.1 g
